# Advances in Pediatric Diagnostic Endoscopy: A State-of-the-Art Review

**DOI:** 10.1097/PG9.0000000000000224

**Published:** 2022-07-29

**Authors:** Diana G. Lerner, Ali Mencin, Inna Novak, Clifton Huang, Kenneth Ng, Richard A. Lirio, Julie Khlevner, Elizabeth C. Utterson, Brendan R. Harris, Ryan T. Pitman, Sabina Mir, Roberto Gugig, Catharine M. Walsh, Doug Fishman

**Affiliations:** From the * Department of Pediatrics, Division of Pediatric Gastroenterology, Hepatology and Nutrition, Medical College of Wisconsin, Milwaukee, WI; † Division of Pediatric Gastroenterology, Columbia University Vagelos College of Physicians and Surgeons, New York, NY; ‡ Department of Pediatrics, Division of Pediatric Gastroenterology, Hepatology and Nutrition, Children’s Hospital at Montefiore, Bronx, NY; § Department of Pediatrics, Division of Pediatric Gastroenterology, Hepatology and Nutrition, Cook Children’s Medical Center, Fort Worth, TX; ∥ Division of Pediatric Gastroenterology, Hepatology and Nutrition, Johns Hopkins University School of Medicine, Baltimore, MD; ¶ Department of Pediatrics, Division of Pediatric Gastroenterology, Hepatology and Nutrition, UMASS Memorial Children’s Medical Center/UMASS Medical School, Worcester, MA; # Department of Pediatrics, Division of Pediatric Gastroenterology, Hepatology and Nutrition, Washington University School of Medicine, St. Louis Children’s Hospital, St. Louis, MO; ** Department of Pediatrics, Division of Pediatric Gastroenterology, Hepatology and Nutrition, UNC School of Medicine, Chapel Hill, NC; †† Lucile Packard Children’s Hospital at Stanford, Palo Alto, CA; ‡‡ Department of Paediatrics and the Wilson Centre, Division of Gastroenterology, Hepatology and Nutrition and the Research and Learning Institutes, Hospital for Sick Children, University of Toronto, Toronto, Canada; §§ Department of Pediatrics, Division of Pediatric Gastroenterology, Hepatology and Nutrition, Baylor College of Medicine, Houston, TX.

**Keywords:** endoscopy, therapeutic endoscopy, pediatric, confocal laser endomicroscopy, endoflip, wireless motility capsule, chromoendoscopy, EUS, optical coherence tomography, colon capsule

## Abstract

Pediatric endoscopy has revolutionized the way we diagnose and treat gastrointestinal disorders in children. Technological advances in computer processing and imaging continue to affect endoscopic equipment and advance diagnostic tools for pediatric endoscopy. Although commonly used by adult gastroenterologists, modalities, such as endomicroscopy, image-enhanced endoscopy, and impedance planimetry, are not routinely used in pediatric gastroenterology. This state-of-the-art review describes advances in diagnostic modalities, including image-enhanced endoscopy, confocal laser endomicroscopy, optical coherence tomography, endo functional luminal imaging probes, wireless motility/pH capsule, wireless colon capsule endoscopy, endoscopic ultrasound, and discusses the basic principles of each technology, including adult indications and pediatric applications, safety cost, and training data.

**What is Known**

- Application of novel technology in diagnostic endoscopy in pediatrics is lagging.

**What Is New**

- Description of chromoendoscopy, optical coherence tomography, colon capsule, wireless motilty/pH capsule, Endoflip, Endomicroscopy, EUS as they relate to advances in diagnostic endoscopy in children.

## INTRODUCTION

From its inception in the 1970s, pediatric endoscopy has revolutionized the way we diagnose and treat gastrointestinal (GI) disorders in children. Technological advances in computer processing and imaging continue to affect endoscopic equipment and advance diagnostic tools for pediatric endoscopy. Although commonly used by adult gastroenterologists, modalities such as endomicroscopy, image-enhanced endoscopy, and impedance planimetry, are not routinely used in pediatric gastroenterology.

The North American Society for Pediatric Gastroenterology, Hepatology and Nutrition (NASPGHAN) Endoscopy and Procedures Committee presents the first article in the series of *state-of-the-art* technology reviews. This article will focus on advances in diagnostic tools for pediatric gastrointestinal disorders. Although some technologies discussed in this article such as image-enhanced endoscopy, endomicroscopy, and optical coherence tomography are only beginning to carve their role in pediatric GI disorders, indications for endoscopic ultrasound are rapidly expanding. Each section will describe the basic principles of the technology, adult indications, and pediatric applications, and provides safety, cost and training data when available. The committee hopes that familiarly with newer technology will allow the pediatric gastroenterologist to apply these tools for the benefit of our young patients.

## METHODS

The North American Society for Pediatric Gastroenterology, Hepatology, and Nutrition (NASPGHAN) Endoscopy Committee identified a need to review new diagnostic modalities in pediatric endoscopy and their potential applications in diseases during childhood.

Each section was written by pediatric endoscopists who conducted literature reviews focusing on all available published pediatric experience. The search was conducted using the PubMed database from 2016 to 2021. Some of the sections were based on expert opinion due to paucity of pediatric data. The topics selected were proposed to the NASPGHAN Council and were approved. Images provided are the authors own (pediatric) or were supplied by the manufacturer (adult). We thank Dr Charles Lightdale and Dr Jenifer Lightdale for their expertise and editing.

## ADVANCES IN PEDIATRIC DIAGNOSTIC ENDOSCOPY

### Image-Enhanced Endoscopy

Image-enhanced endoscopy refers to the use of dye or enhanced optics and color lenses to optimally delineate minor changes in the mucosa of the GI tract. Although this concept has existed since the 1970s, the technology and its applications continue to expand. The enhanced images have improved the diagnostics for Barrett’s esophagus, adenocarcinoma, *H. pylori*, and colon cancer surveillance.

#### Description of Technology

Chromoendoscopy is a technique during which the endoscopist uses a spray catheter to paint intestinal mucosa with a dilute dye agent to enhance visualization of mucosal abnormalities. Dye-based endoscopy increases diagnostic yield for detection of mucosal irregularity, including Barrett’s esophagus, dysplasia, and neoplasia in high-risk individuals. Use of dye enhances diagnostic yield of biopsies by improving visualization of microstructure and vascular patterns of the lesions under investigation and delineating the boundaries between normal and abnormal mucosa. Various stains exist and are classified according to their mechanism of action into contrast, absorptive, and reactive stains. Contrast and absorptive stains are best used for evaluating for malignancy or inflammation. Reactive stains change colors based on chemical reaction and are used primarily for *H. pylori* detection or diagnosis of hypochlorhydria. The American Society for Gastrointestinal Endoscopy (ASGE) technology status evaluation report summarizes the performance of chromoendoscopy, stratified by stain type (Table 1 adapted from the ASGE report) (1–3).

**TABLE 1.** Description of contrast dye for chromoendoscopy

Stains		Visual Effect/Action	Main Application	Technique
**Absorptive stains**	Lugols solution (iodine + potassium iodide)	Glycogen-containing normal squamous epithelium stains dark brown. Lack of staining suggests abnormality.	Barrett’s esophagus Esophageal cancer	Spray 20–30 mL 1.5–3% dye to esophageal mucosa.
	Methylene blue	Barrett’s mucosa stains dark blue despite irrigation, inconsistent blue staining suggests dysplasia, and cancer	Barrett’s esophagus, gastric intestinal metaplasia, cancer, chronic UC	Pretreat with mucolytic agent Spray 0.5% dye, dwell 1–2 min wash vigorously. For pan colonic staining use 0.1% dye to spray 20–30 cm, dwell 1 min, wash and evaluate. Methylene blue-MMX tablets
	Crystal violet	Absorbed into intestinal and neoplastic cells	Barrett’s esophagus, colonic neoplasms	Use 0.05–0.1% dye similar to methylene blue.
	Toluidine blue	Nuclei of malignant cells are stained blue. False positive can occur with inflammatory and fibrotic lesions	Oral and esophageal squamous cell cancer	Prewash with 1% acetic acid, apply 10–20 mL of 1% solution, after 1 min, rewash with acetic acid.
**Contrast stains**	Indigocarmine	Blue dye helps delineate topography of the mucosa and pit pattern.	Gastric lesions, colonic neoplasms, chronic UC	Use 0.1–0.8% dye, immediately evaluate for mucosal irregularity/pit pattern.
**Reactive stains**	Congo red	Color change from red to dark blue/black in presence of acid at pH < 3	Ectopic gastric mucosa, gastric cancer, vagotomy evaluation	Give secretagogue (eg, pentagastrin 5 m g/kg). Rinse mucosa with 0.5–5% sodium bicarbonate solution. Spray 0.3–0.5% congo red. Acid-secreting areas become black within minutes.
	Acetic acid	Color change from yellow to red in presence of alkali	Barrett’s esophagus	Dilute acetic acid (3%) is utilized primarily to detect dysplasia in Barrett’s esophagus. It is widely available and inexpensive. It creates a white enhanced surface pattern (acetowhite) with dysplastic areas appearing more red.

White light endoscopy (WLE) uses a xenon light source which has a broad wavelength (400–700 nm) and produces rosy images due to reflection of red wavelengths. It is the standard of care for diagnosis and evaluation of the GI tract but has some limitations in identifying subtle musical changes.

Electronic chromoendoscopy (EC) utilizes electronic image processing to enhance visualization of certain mucosal features allowing for enhancement of tissue structures. It is easily available to the endoscopist by pressing a button on the currently available endoscopes alleviating the need to use catheters or dyes. EC technology includes narrow-band imaging (NBI) (Olympus Medical Systems, Tokyo, Japan), flexible spectral imaging color enhancement (FICE) (Fujinon, Fujifilm Medical Co, Saitama, Japan), and i-SCAN (PENTAX Endoscopy, Tokyo, Japan) (4) (Table 2). Current indications include evaluation of gastroesophageal reflux disease (GERD), Barrett’s esophagus, gastric neoplasia, and polyp classification.

**TABLE 2.**
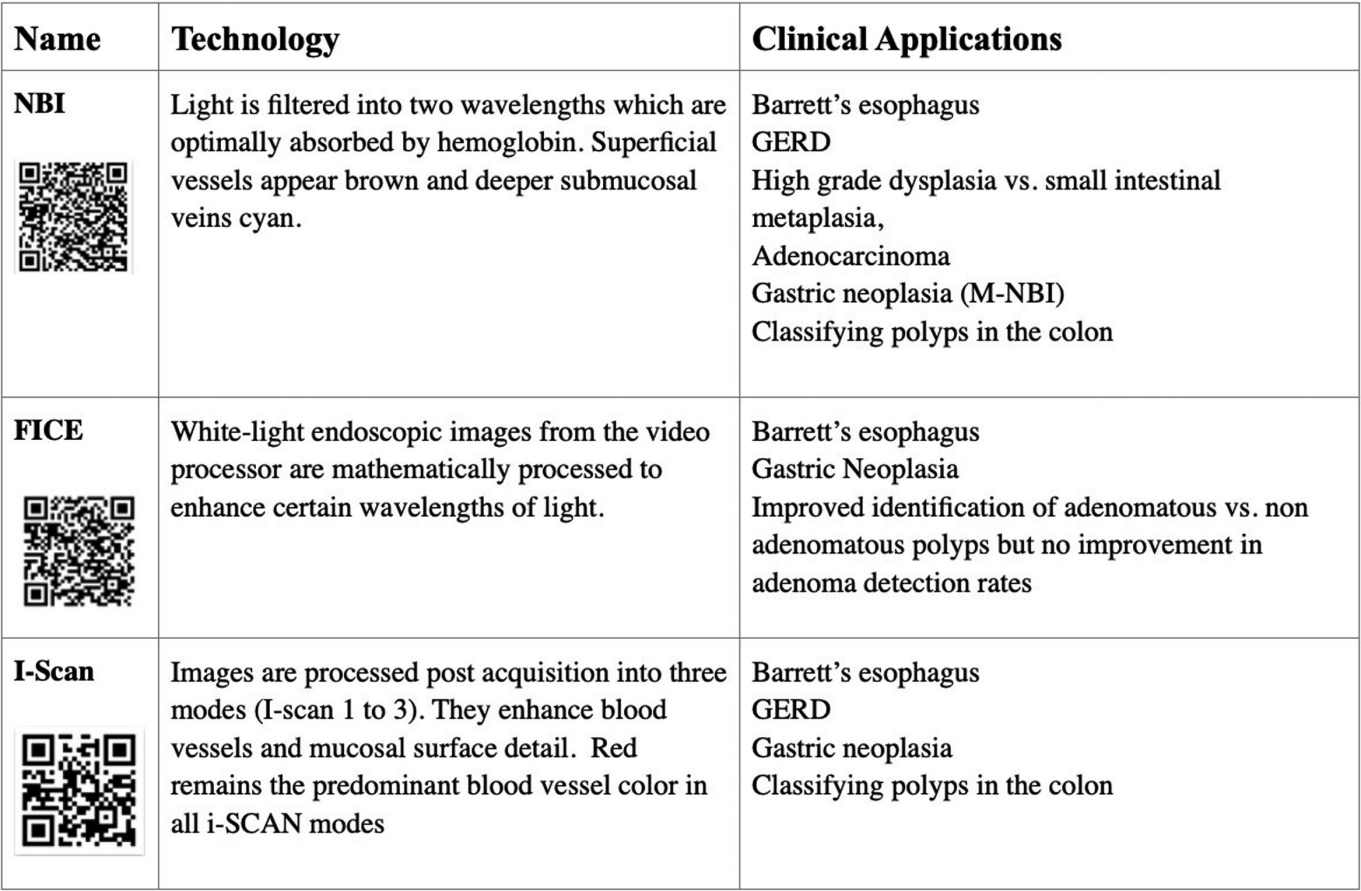
Electronic chromoendoscopy technologies

NBI is an optimal imaging technology that enhances visibility of vessels and other structures on or near the mucosal surface, FICE converts images into spectral images with individual wavelengths and reconstructs using spectral estimation process to generate fine high contrast images. i-Scan uses algorithms to enhance tissue differences based on unique reflective properties in healthy and diseased tissue (5).

#### Adult Indications

Adult indications for image-enhanced endoscopy (IEE) are outlined in Table 2. Dye-based IEE endoscopy has been studied in the esophagus, stomach, and colon. Image processing is superior to white light endoscopy for the diagnosis of Barrett’s esophagus, GERD, gastric metaplasia, and adenocarcinoma (5). Lugol iodine when sprayed on esophageal tissue with squamous cell carcinoma and high-grade dysplasia will leave this area unstained making it easier to identify over white light. Previously, acetic acid application was also shown to be highly sensitive and specific for the diagnosis of early esophageal cancer and high-grade dysplasia (6), whereas Barrett’s esophagus was better visualized after methylene blue application. For the detection of high-grade dysplasia, NBI and acetic acid chromoendoscopy met the ASGE established performance thresholds (sensitivity of 90%, specificity of 80%, and negative predictive value of 98%) (7).

In the stomach, indigo-carmine’s blue dye can delineate tumor margins and abnormal tissue, whereas acetic acid and indigo-carmine mixtures and methylene blue improve the detection of premalignant lesions (2). In the colon, indigo-carmine increased adenoma detection rates especially for smaller, flat, or depressed lesions.

Colorectal cancer in patients with inflammatory bowel disease (IBD) develops from dysplastic changes in the epithelium that progress to malignancy (8). Current recommendations suggest conducting screening colonoscopies every 1–2 years, starting 7–10 years after diagnosis in patients with ulcerative colitis and colonic Crohn’s disease (9–11). Yearly screenings starting 1–2 years after diagnosis are recommended in those with primary sclerosing cholangitis, due to increased risk of colorectal cancer and the high rate of progression from low-grade dysplasia (9,11,12). Surveillance for Colorectal Endoscopic Neoplasia Detection and Management in Inflammatory Bowel Disease Patients: International Consensus Recommendations (SCENIC) published a consensus statement in 2015 advocating for the use of chromoendoscopy over white light endoscopy for surveillance of cancer in IBD. This recommendation was based on a prospective, tandem study which found a significant increase in diagnosis of lesions after the colonoscopy was repeated in 75 patients using chromoendoscopy (10). Chromoendoscopy increased the rate of detection of dysplastic lesions by 2- to 3-fold compared with white light endoscopy (13,14). The procedures were on average 11 minutes longer when compared to white light endoscopy (15). NBI was not shown to be superior in surveillance endoscopy for ulcerative colitis patients; however, classifications of polyps as adenomas in real time is improved with NBI filter, especially after endoscopists complete computer-based training (16). Fewer studies are available for i-SCAN and FICE, but comparative studies between the three technologies show no difference (4).

#### Pediatric Applications

There is a paucity of data about pediatric applications for IEE. However, predisposing conditions put children at increased risks for early onset adult diseases. For instance, the incidence of Barrett’s esophagus in children with no neurodevelopmental or tracheoesophageal conditions is only 1.43% (17) but increases to 15% (1.3–15%) in children with esophageal atresia (18,19). Although adenocarcinoma is rare in the pediatric age range (20), early detection of esophagitis allows for optimization of therapy and risk mitigation. Barrett’s esophagus confers a 40- to 50-fold increased risk of developing adenocarcinoma over the general population (7).

A study on patients with PSC-UC/IBD-U highlights the importance of screening for colorectal cancer (CRC) in pediatric patients. The authors reviewed data for 509 patients (median age of IBD diagnosis was 12.6 years) and the 5-year probability of developing CRC after diagnosis was 0.8% and 10-year probability was 4.8%. A recent meta-analysis by El-Matary and Bernstein highlights the increased cancer risk in pediatric IBD and very early onset IBD, recommending surveillance and possible revision of current surveillance guidelines (21). Chromoendoscopy or IEE can become an important tool for pediatric gastroenterologists performing screening for Barrett’s esophagus or CRC in high-risk populations.

#### Additional Information

##### Safety

Special attention should be paid during dye-based chromoendoscopy to avoid pooling of the dyes during procedure, as it can limit evaluation of the underlying mucosa. Concerns about DNA damage with the use of methylene blue have been raised (22), but an oral dose of 200 mg of methylene blue-multimatrix did not result in any detectable DNA damage (23). Lugol iodine may lead to chest discomfort and nausea and on rare occasions can cause esophagitis or gastritis.

##### Costs

Overall equipment costs are minimal. Chromoendoscopy required dye and special spray catheters. Spray catheters are $60–100. Most dyes range in cost between $25 and $150.

##### Training

Several resources are available for self-learning in chromoendoscopy, including videos, books, and web-based educational materials (for example, ASGE training video available at https://www.youtube.com/watch?v=OARkbgwlObI). Improvement in lesion detection has been shown even in novice users following training (2).

### Confocal Laser Endomicroscopy

Confocal laser endomicroscopy (CLE) was developed to obtain high magnification and resolution images known as “optical biopsies” of the GI mucosal histology at the cellular and sub-cellular levels (24–26). The advantage of CLE is that normal tissue can be identified with high accuracy and confidence during endoscopy. If real-time histologic evaluation is done by an expert, reliance on random biopsies in diseases, such as ulcerative colitis is decreased. Microscopically targeted “smart” biopsies with higher yield result in needing fewer samples (27,28). Since its first description in 2004, the number of diseases studied with this technique has steadily grown (29). Organs of interest for CLE include luminal structures (esophagus, stomach, and colon), ductal structures (bile and pancreatic ducts), and parenchymal structures (liver) (25). The technique has also been applied in other specialties including pulmonology (30), urology (26), and gynecology (31).

#### Description of Technology

Unlike conventional endoscopy, which uses white light and lenses to magnify an image, CLE employs a low-power laser which is focused on a selected depth in the tissue of interest. The fluorescence of light reflected from the tissue is subsequently focused through a pinhole. The light is then detected by a photodetector and transformed into an electrical image by a computer system, which creates a gray-scale representation of one specific plane. The term confocal refers to the alignment of both illumination and collection systems in the same focal plane. The magnification of the mucosal tissue is 1000-fold, with sequentially deeper images from the epithelial surface to approximately 250 μm below the surface.

**FIGURE 1.**
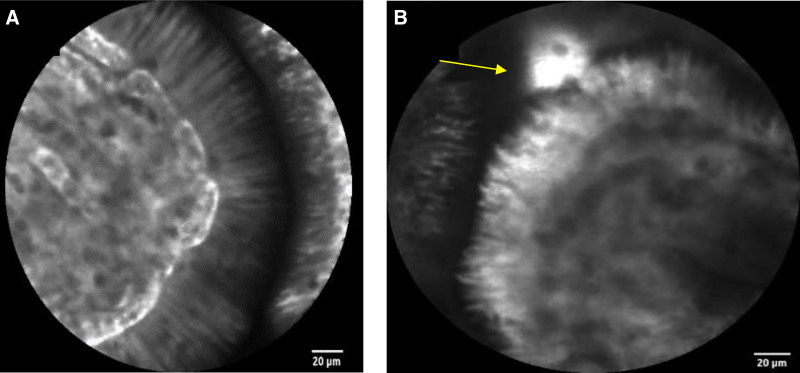
CLE of the terminal ileum. (A) Normal epithelial barrier and no extravasation of fluorescein is seen. In (B), a dysfunctional epithelial barrier with extravasation of fluorescein (yellow arrow). CLE = confocal laser endomicroscopy.

Confocal imaging can be based on tissue reflectance or fluorescence (32,33), which does not require contrast agents; however, current prototypes have relatively low resolution and limited clinical utility (32). The addition of topical or intravenous fluorescence contrast agents generates images with comparable resolution to traditional histological examination (Fig. 1) (33,34). The fluorescent contrast agents most commonly used for CLE can be administered intravenously (fluorescein sodium, AK-Fluor; Akorn Pharmaceuticals, Lake Forest, United States) or topically (Acriflavin; Sigma Pharmaceuticals, Clayton, Australia), tetracycline, or cresyl violet (AnaSpec Inc, San Jose, United States) through a spraying catheter (35). Intravenously delivered fluorescein distributes throughout the extracellular matrix of the surface epithelium and lamina propria but does not stain cell nuclei (36). Topically administered acriflavin stains cell nuclei of the surface epithelium but does not penetrate deeper layers of the GI mucosa. Fluorescein is usually administered immediately before imaging. Optimal images are obtained within 30 seconds to 8 minutes after injection but can be interpreted for as long as 60 minutes (35). Doses as high as 10 mL of 10% fluorescein have been evaluated, with optimal images obtained after administration of 2.5 to 5 mL (37). After contrast administration, the tip of the confocal endomicroscope or miniprobe is positioned in gentle but firm contact with the area of interest to obtain high-resolution confocal images. Accumulated images can be saved for postprocedural analysis.

Two kinds of CLE systems have been developed: endoscope-based CLE (eCLE) and probe-based CLE (pCLE). In eCLE, CLE is integrated in the tip of a dedicated endoscope, whereas in pCLE a probe is inserted through the accessory channel of a conventional endoscope. eCLE is advantageous as it allows for higher resolution images, a larger field of view, and an adjustable image depth. However, eCLE systems have not been commercially available since 2014. The ability to remove a probe without exchanging the endoscope makes pCLE better for targeted evaluation of lesions and therapeutic interventions, such as endoscopic mucosal resection. The probe-based system to date has a fixed focal length and so it can only scan in a single plane, unlike current microscope systems that can create cross-sectional images at different depths. Transparent caps can be employed at the tip of the endoscope to increase probe stability (25).

#### Adult Indications

##### Upper Gastrointestinal Tract

In the esophagus, the main indication for CLE is for the evaluation of Barrett’s esophagus (38). In cases of dysplasia or neoplasia, the CLE probe has high specificity but poor sensitivity, which may be improved with recent advances in machine learning. Another potential application may be to differentiate Non-Erosive Reflux Disease from normal tissue by identifying microalterations of the esophageal tissue not seen on standard biopsies (39).

In the stomach, CLE was reported to have 93% accuracy for the diagnosis of Helicobacter pylori infection (40). CLE can evaluate for metaplasia, including evaluation of positive lateral margins postendoscopic mucosal resection (EMR) and for gastric adenoma (41). In the small bowel CLE can be utilized to evaluate for villi blunting and architectural distortion that may support the diagnosis for celiac disease. Furthermore, Coron et al, evaluated 15 patients with suspected GI-graft versus host disease and found the sensitivity of probe-based CLE to be 87.5% (42) Fritscher-Ravens described utility of pCLE in evaluation of real-time dynamic barrier changes in 108 patients with irritable bowel syndrome and negative food allergy testing. Positive +pCLE was correlated with mucosal response to foods (predominantly wheat) and microscopic evidence of inflammation in up to 50% of the patients. They hypothesized that in many patients, IBS could be a sign for non-typical food allergy (43).

##### Lower Gastrointestinal Tract

CLE can be utilized to assess Polyps and Neoplasia by classifying and differentiating polyps and assess residual tissue post EMR of neoplastic lesions (44,45). For management of inflammatory bowel disease, CLE can aid in differentiating between active and quiescent disease, assessing the extent of disease, targeting biopsies to evaluate for earlier detection of dysplasia, assessment of mucosal healing and defining treatment protocols (24).

##### Biliopancreatic Systems

CLE has been utilized to evaluate biliary strictures via a dedicated probe passed through a cholangioscope or catheter for ERCP (24). These images provide real-time microscopic images of the biliary epithelium, providing histological information that is not otherwise available during ERCP. At the pancreas, imaging of organs within or adjacent to the GI tract with a miniprobe passed through an endoscopic needle provides in vivo real-time microscopy during EUS which potentially allow for better differentiation of various types of pancreatic lesions (24).

#### Pediatric Applications

Few studies have shown the utility of this technology in pediatric patients. Venkatesh et al described a series of 44 pediatric patients who underwent CLE for GERD, Barrett’s esophagus, peptic ulcer disease, celiac disease, diarrhea, hematochezia, concern for familial adenomatous polyposis with APC mutation, and graft versus host disease. The youngest patient was 8 months old with graft versus host and the smallest patient to tolerate pyloric intubation was 18-month-old and 11 kg. No adverse events were reported (46). This study was done with Pentax EC3870 CILK endoscope, which is not currently commercially available. Shavrov et al studied 24 pediatric IBD patients with serial endoscopy +pCLE. Abnormal findings in TI with pCLE (increased epithelial gap density) was predictive of disease relapse (47).

Hypothetical applications include evaluation for target biopsies in eosinophilic esophagitis (EoE), celiac disease, familial adenomatous syndrome, and allergic colitis. Current adult and pediatric applications are shown in Table 3.

**TABLE 3.** Current adult and pediatric applications of CLE

Organ	Adult applications	Pediatric applications
Esophagus	Barrett’s esophagus	GERD EOE
Stomach	*Helicobacter pylori* infection Gastric metaplasia Evaluation of positive lateral margins post endoscopic resection	*Helicobacter pylori* infection
Small intestine	Celiac disease Inflammatory bowel disease Irritable bowel disease Atypical food allergies	Celiac disease, inflammatory bowel disease
Large intestine	Polyps and neoplasia Inflammatory bowel disease Graft versus host disease	IBD Genetic polyposis syndromes Allergic colitis Graft versus host disease
Biliary	Evaluation of lesions, strictures	Evaluation of lesions, strictures
Pancreas	Evaluation of lesions	Evaluation of lesions

CLE = confocal laser endomicroscopy; EOE = eosinophilic esophagitis; GERD = gastroesophageal reflux disease.

#### Additional Information

##### Safety

CLE is a safe procedure. Mild adverse effects are reported in up to 1.4% of patients and include nausea and vomiting, transient hypotension, rash, epigastric pain, and erythema at the injection site. More serious side effects can include allergic reactions to the dye, including anaphylaxis, seizure, shock, and cardiac ischemia. The safety of topical stains/dyes requires further study. Acriflavin and Cresyl violet are not approved by the FDA. Some dyes are known mutagens and potential carcinogens (48).

##### Cost

The high cost of CLE equipment remains a limitation of this technology, and probes have a limited number of clinical uses. Currently available pCLE probes and systems and eCLE endoscopes are outlined in Table 4. Before the CLE can be widely accepted, the efficacy and cost-effectiveness of the technology compared with other available advanced imaging technologies require further study.

**TABLE 4.** Currently available CLE equipment

	Compatible operating channel	Length, m	Max No. uses	Field of view diameter	Probes	Compatible operating channel	Cost
**Probes**							
GastroFlex UHD	≥ 2.8 mm	3	20	240	1.0	55–65	$9200
CholangioFlex	≥1.0 mm	4	10	325	3.5	40–70	$9900
AQ-Flex 19	≥ 0.91 mm (19 gauge)	4	10	325	3.5	40–70	$8900
ColoFlex UHD	≥ 2.8 mm	4	20	240	1.0	55–65	$9900
**System**							
Cellvizio 100 series system^1^	–	–	–	–	–	–	$175 000

^1^Mauna Kea Technology, Paris, France.

CLE = confocal laser endomicroscopy.

##### Training

Training in CLE is typically acquired through continuing medical education courses, visitation with experts, and online resources. A comprehensive library of pCLE case studies and an image atlas is available online (www.cellvizio.net) and through a Smartphone application (from www.cellvizio.net). Training for eCLE is typically offered through experienced centers. As with any new diagnostic tool, a learning curve exists with respect to proper probe targeting, image interpretation, and accurate diagnosis using CLE. The extant literature is sparse; however, the few reported studies indicate a short learning curve (49–51), typically aided by review of standardized image libraries (49). In a study by Buchner et al involving 11 endoscopists with varied levels of pCLE experience, diagnostic accuracy in the evaluation of 76 colorectal polyp pCLE sequences rose from 63% during interpretation of the first 20 images to 86% during interpretation of the final set of images (49). Inter- and intraobserver agreement with respect to the GI tract has been reported as good to excellent(50,52,53).

### Optical Coherence Tomography

Optical coherence tomography (OCT) was developed for noninvasive cross-sectional imaging. Since its development OCT has been widely used in clinical and pathological applications in a variety of fields, such as ophthalmology, cardiology, gastroenterology, pulmonology, and oncology. OCT can provide broad field, sub-surface, near microscopic imaging during endoscopy. It is particularly useful in evaluation of villous morphology and first layers of the gastrointestinal wall (54).

#### Description of Technology

OCT uses low-coherence interferometry to produce a two-dimensional image from internal tissue microstructures and is analogous to ultrasound, except OCT uses light instead of sound to produce images. Optical signal that is transmitted through or reflected from biological tissue contains time-of-flight information, which in turn yields spatial information about tissue microstructure. Tomographic imaging techniques, such as x-ray computed tomography or magnetic resonance imaging and ultrasound imaging, have found many applications in medicine. “Coherence” refers to a temporal property of light. Low-coherence interferometry enables measurement of echo delay and magnitude of backscattered light from internal tissue microstructures. Initially described in 1991, OCT has involved overtime to have rapid imaging and higher resolution (55). Fourier domain detection enabled real-time 3D-OCT imaging for in vivo biomedical applications with a resolution of 5–10 µm. A limitation of OCT includes the need for standardized terminology and criteria for normal and neoplastic tissues.

#### Adult Indications

OCT has been used for imaging of the esophagus, stomach, small and large intestine and with development of smaller probes biliary and pancreatic ducts. Development of endoscopic ablative therapies and endoscopic mucosal resection improved treatment of GI cancers. Early detection of dysplasia and cancers, particularly in the setting of chronic inflammation associated with conditions such as esophagitis and IBD, is crucial to successful treatment of these conditions. OCT can minimize inadequate sampling. A study by Pfau et al showed that adenomas had significantly less structure and scattered light to a lesser degree than hyperplastic polyps (56). OCT has been characterized in the normal colon, ulcerative colitis, and Crohn’s disease (57). Depth of images obtained by OCT can help differentiate the transmural inflammation of Crohn’s disease from ulcerative colitis (58). As the OCT probes became smaller, it became possible to use this technology for imaging of biliary and pancreatic ducts and evaluate strictures for neoplasia during endoscopic retrograde cholangiopancreatography (ERCP). In 2001, Seitz and colleagues first demonstrated the layered architecture of the in vivo bile ducts similar to that found on histologic sections as well as retroperitoneal structures with less backscattering (59). OCT can differentiate chronic pancreatitis from normal biliary duct and from neoplastic lesions (54,60).

#### Pediatric Applications

OCT is a promising noninvasive imaging technology easily accessible through the working channel of an endoscope and has been performed in various parts of the GI tract. A prospective, blinded study by Shen and colleagues (58) showed a sensitivity of 90.0% and specificity of 83.3% for OCT in detecting the disrupted layered structure of the colon wall indicative of transmural inflammation, providing a valuable tool to distinguish Crohn’s disease from ulcerative colitis, which is particularly useful in pediatric patients with indeterminate colitis. Although OCT has a more limited imaging depth as compared to endoscopic ultrasound, it enables an endoscopist to study the microstructure of the first layers of the intestine and might be useful in differentiation of benign and malignant lesions, especially in the setting of inflammation. This technology can be utilized to identify malignant transformation in polyposis disorders. Masci et al (61) reported on the use of OCT during real-time endoscopic imaging for the evaluation of duodenal villous morphology. OCT and histology showed total concordance for the evaluation of villous morphology in patients with and without celiac disease. OCT was also able to different degrees of villous atrophy. With further refinement of this technology, OCT may allow for “true optical biopsies” in the future.

#### Additional Information

##### Safety

No adverse events are reported.

##### Cost

Use of OCT is mainly restricted to major academic and research institutions, owing to the cost of equipment. One of the more recent commercially available or custom-made OCT probes, the NvisionVLE Imaging System (Nine Point Medical, Cambridge, United States), costs approximately $200–235k USD.

##### Training

Training is provided by the manufacturer after equipment purchase.

### Endo Functional Luminal Imaging Probe

#### Description of Technology

The Endo Functional Luminal Imaging Probe 1.0 system (EndoFLIP); Medtronic, Minneapolis, United States) utilizes high-resolution impedance planimetry to assess the GI lumen. The balloon catheter contains 16 impedance sensors spaced either 0.5 or 1 cm apart, and a solid-state pressure transducer at the distal end of the catheter. Volume-controlled distension of the balloon catheter using a saline-based conducive fluid from the provided syringe produces luminal data in real time on a standalone computer screen. Information provided includes the diameter, cross-sectional area, compliance, pressure, and distensibility index (calculated based on the smallest cross-sectional area divided by the balloon pressure). The 2.0 system includes an additional FLIP topography computer that converts data from the 1.0 system and displays it relative to time. When used in the esophagus, this upgrade assesses motor functions and complements high-resolution manometry. Repetitive anterograde contractions represent normal esophageal motor functions in response to volume distension. Abnormal contractions (such as repetitive retrograde contractions) or the absence of contractions may suggest motor-based disorders. To counteract pressure variances associated with normal physiologic processes and esophageal contractility, software is available from the manufacturer and other sources to aid with analysis. No standardized pediatric protocol or pediatric reference ranges are reported. Multicenter prospective pediatric studies are needed to define these parameters. Although this system measures the diameter and cross-sectional area, it may not identify asymmetric segments (i.e., due to prior surgical interventions). Limitations of the technology exist for evaluation of sphincters after surgery, but pre/post measurements may be beneficial. Lack of smaller balloons may be an issue in certain populations.

#### Adult Indications

Clinical use of this technology is evolving and now includes evaluation of the GI tract (62) (Table 5). Indications for EndoFLIP include measurement of pressure and dimensions in the esophagus, stomach, and anus, as an adjunctive tool for patients with GI motility disorders and intraoperatively to assist with esophageal and bariatric procedures (57) (Table 5).

**TABLE 5.** Current proposed adult and pediatric applications of EndoFLIP

Organ	Adult and pediatric applications
Oropharynx	Measurement of upper esophageal sphincter compliance Evaluation of pharyngoesophageal segment tone and swallowing
Esophagus	Achalasia, evaluation of gastroesophageal junction pressure after POEM or Heller myotomy, fundoplication, Eosinophilic esophagitis, Evaluation of postfundoplication dysphagia
Stomach	Pyloric compliance ingastroparesis, refractory nausea/vomiting Measurement of response after pyloric dilation Adjunct to hiatal hernia repair Adjunct to a bougie for measuring the size of a gastric sleeve created during bariatric surgery
Large Intestine	Fecal incontinence

EndoFLIP = endo functional luminal imaging probe; POEM = peroral esophageal myotomy.

Best practice advice recently published by the American Gastroenterological Association Institute on the utilization of EndoFLIP suggests it can be used as a complementary diagnostic tool for measurement of esophageal junction opening dynamics and esophageal wall stiffness (63). They do not advocate using the probe for routine diagnosis of GERD or EoE but they do suggest that it may be clinically useful in assessment of disease severity and therapeutic monitoring in patients with EoE.

The esophagogastric junction distensibility index can be used to objectively gauge luminal change before and after endoscopic or surgical interventions (ie, peroral endoscopic myotomy, Heller myotomy, or fundoplication) (64–66). For myotomies, an increase in the distensibility index should be seen. Conversely, a decrease in the distensibility index is expected for fundoplication. The normal distensibility index of the adult patient’s esophagogastric junction is greater than 2.8 mm^2^/mmHg, at 60 mL inflation (67). Similarly, EndoFLIP can measure the diameter and cross-sectional area at a stenotic region before and after dilation (balloon or mechanical), confirming luminal response to the intervention. This technology can also be used in patients with suspected fecal incontinence (62) and was shown to be >70% concordant with high-resolution anorectal manometry to identify anal deficiency in one adult study. The EndoFLIP 2.0 system may be used to complement manometry studies when evaluating patients for esophageal dysmotility disorders, including achalasia (68,69).

#### Pediatric Applications

EndoFLIP was approved by the United States Food and Drug Administration (FDA) for assessment of the esophagus, pylorus, and anal sphincters in children 5 years of age and older in early 2019. Published data have shown successful off-label use of EndoFLIP in children as young as 10 months of age (70). It has been described in the assessment of luminal distensibility in pediatric patients with and without EoE (71). Ng and colleagues reported successfully using EndoFlip to guide esophageal dilation in 19 children (72). EndoFLIP is now routinely used to evaluate success in achalasia surgery in both POEM and post Heller Myotomy in children (73).

#### Additional Information

##### Safety

No adverse events are documented in either adult or pediatrics.

##### Cost

Details on costs associated with the EndoFLIP are available in Table 6.

**TABLE 6.** Cost of endo functional luminal imaging system (EndoFLIP)

Device	Model	Cost $
EndoFLIP	EF-100 (1.0 system)	24 058
EndoFLIP	EF-100 (1.0 system) plus DD-961 topography add-on (= 2.0 system)	69 500
Digital Media Recorder (optional)	Tokyo Electro Acoustic Company D-971	6000
Diagnostic Catheters	EF-322 (16 cm catheter) EF-325 (8 cm catheter)	1725 per box of 5 catheters

##### Training

Medtronic provides training and suggest the typical learning curve is 4–5 endoscopy cases. Recent retrospective review found that overall procedural time with functional luminal imaging probe decreases with additional cases performed (68).

### Wireless Motility/PH Capsule

#### Description of Technology

The wireless motility/pH capsule (WMC; SmartPill Wireless Motility Capsule, SmartPill Corporation; Buffalo, United States) is an orally ingested, nondigestible, data recording device measuring approximately 26.8 mm in length and 12 mm in diameter (slightly larger than a multivitamin) that provides real-time measurement of the temperature, pH, and pressure, which enables the assessment of gastric emptying time, small bowel transit time, colonic transit time, and whole gut transit time without radiation exposure (74). The capsule consists of a rigid polyurethane shell containing a battery that lasts for a minimum of 120 hours, sensors for pH (range, 0.05–9.0), temperature (range, 25–49°C), and pressure (range, 0–350 mmHg), and a transmitter that operates at a wavelength of 434 MHz (75). Patients are provided with a SmartBar standardized meal to eat along with 50 mL of water before swallowing the WMC. Before ingestion, the capsule requires activation using a magnetic fixture followed by pH calibration using the provided buffer. To obtain an accurate measurement of gastric emptying time, the patient must wait 6 hours after ingesting the capsule before eating another meal.

As the capsule passes through the GI tract, miniaturized sensor technology measures pressure, temperature, pH, real, and elapsed time. Acquired data are continuously transmitted over very low-power radiofrequencies to a small receiver that can be worn on the patient’s belt for 3–5 days. Although the capsule normally has a transit time ranging from 24 to 48 hours, it is capable of transmitting data continuously up to 5 days in patients, useful in those with reduced motility. Patients are instructed to push the event button and to keep a diary of events (eg, meals, sleep, bowel movements) during the duration of the study. Once the capsule has passed, the data set is downloaded from the receiver to a laptop computer, and special software provides tools for data analysis and a graphical user interface that indicates when gastric emptying, small bowel/large bowel transit, and total GI tract transit time of the capsule has occurred (75).

#### Adult Indications

The FDA approved WMC for the evaluation of adult patients with suspected delayed gastric emptying (gastroparesis) in 2006 and for the evaluation of colonic transit in adult patients with chronic idiopathic constipation in 2009. In adult clinical trials, the sensitivity, specificity, and receiver-operating characteristics of WMC are comparable with those of radiopaque marker tests, antroduodenal manometry, and scintigraphic gastric emptying (76); WMC has also been validated in a large clinical trial of subjects with constipation (77,78). Diagnostic utility of wireless motility capsule in adult patients with suspected GI dysmotility has also been examined. WMC confirmed clinical suspicion, provided new diagnostic information, influenced clinical management, and detected a generalized motility disorder in many patients with good device agreement with conventional tests (79).

#### Pediatric Applications

To date, experience in the use of WMC in pediatrics is limited. One study comparing scintigraphic gastric emptying and antroduodenal manometry studies with WMC in symptomatic pediatric patients concluded that the WMC is highly sensitive compared with scintigraphic gastric emptying studies in detecting gastroparesis and more sensitive than antroduodenal manometry in detecting motor abnormalities. The WMC was well tolerated in all subjects without side effects (80). More robust studies with larger numbers of patients with symptoms suggestive of GI dysmotility compared with healthy children are needed to further investigate the correlation between antroduodenal manometry, colonic manometry, and WMC, better understand the significance of WMC findings regarding pathophysiology of the disease and identify the optimal pediatric patient population for this emerging technology.

#### Additional Information

##### Safety

Test failure has been reported for reasons including inability of the patient to swallow the capsule, failure of the capsule to record or transmit data (increased risk in patients with body mass index >40), failure of the receiver to record or download data, and software malfunction. The most serious adverse events associated with WMC are inability to confirm passage of the capsule outside the body, capsule retention, aspiration, and obstruction (74). Magnetic resonance imaging should not be obtained until the passage of WMC is confirmed. Use of smart pill is contraindicated in patients with history of gastric bezoar, swallowing disorders, suspected or known stricture, fistulas or obstruction, history of gastrointestinal surgery within the past 3 months, cardiac pacemakers, or other implanted electromedical devices (74).

##### Cost

The WMC motility monitoring system can be purchased from Given Imaging/Medtronic.

The system is comprised of SmartPill capsules, a SmartPill data receiver, a SmartPill activation fixture, a SmartPill docking station, and a system computer loaded with MotiliGI software. A Smart Pill starter Kit is approximately $20 000 USD plus $3000 for 5 pack SmartPill Capsule/5pack SmartBar.

##### Training

WMC is an office-based standardized technology that is radiation-free and can be readily adopted for clinical use by most gastroenterologists in clinical practice. The manufacturer of WMC provides training upon purchasing of the system.

### Wireless Colon Capsule Endoscopy

#### Description of Technology

Colon capsule endoscopy is a wireless capsule to evaluate the colon. First-generation colon capsule (CCE-1) was introduced in 2006 by Given Imaging (now Medtronic) (Yoqneam, Israel). To improve the accuracy of CCE-1, the second-generation colon capsule (CCE-2) was developed and approved by the FDA in 2014 (81). The CCE-2 is 11.6 × 31.5 mm in size with a battery lasting about 10 hours. It has two cameras, with one at each end, and the capsule is equipped with an adaptive frame rate. This allows each camera to take 35 images per second when in motion and 4 images per second when stationary. The rate of image capture is a result of communication between the capsule and the capsule’s data recorder. The data recorder also stores the capsule’s images (81). Once ingested, the capsule starts taking pictures at 14 images per second until it reaches the small bowel. In the small bowel, it switches to the adaptive frame rate mode. The data recorder can be programmed to give signals to the patient to take preparations per protocol and also informs when the procedure is completed (82). At completion, the data are downloaded from the recorder for viewing (83). The colon capsule requires a bowel preparation. The use of capsule colon endoscopy has advantages over standard colonoscopy. Colon capsule is performed in an unsedated patient and does not require discontinuation of medications, such as anticoagulants. It is advantageous in patients with an incomplete colonoscopy and in patients with contraindications or refusal for conventional colonoscopy.

#### Adult Indications

Conventional colonoscopy represents the gold standard for examination of the colon. According to the European Society of Gastrointestinal Endoscopy (ESGE), CCE can be used for adults who are not at high risk for colon cancer (84). In 2014, FDA approval was granted for CCE based on data from a 16-site clinical trial involving 884 patients that assessed the safety and effectiveness of CCE in detecting adenomas at least 6 mm in size (85).

#### Pediatric Applications

A prospective study conducted by Salvatore et al evaluated the accuracy of CCE-2 in assessing disease activity of the small bowel and colon in 40 pediatric patients with Crohn’s disease (mean age 13.1 ± 3.1 years). The CCE-2 was compared with magnetic resonance enterography, small intestine contrast ultrasonography, and ileocolonoscopy. Sensitivity and specificity of CCE for colon inflammation was 89% and 100%, respectively. Detection of small bowel inflammation had 90% sensitivity and 94% specificity. These were higher than those for magnetic resonance enterography and small intestine contrast ultrasonography (85). Only one pilot study has been performed to date using CCE-2 in pediatric patients with ulcerative colitis. Twenty-nine patients (mean age 14.1 ± 3.2 years) ingested the capsule and underwent a colonoscopy. Sensitivity of CCE-2 in detecting disease activity was 96% (95% CI, 79%-99%) and specificity was 100% (95% CI, 61%-100%). No serious adverse events were reported (86). Future multicenter studies in pediatrics are required to determine the implications of CCE-2. Other possible applications can include polyps screening, follow up of incomplete colonoscopy and evaluation of mucosal healing in IBD.

#### Additional Information

##### Safety

Adverse events are comparable to small bowel capsule endoscopy and include capsule retention and incomplete study. Contraindications include dysphagia, prior major abdominal surgery, bowel obstruction, implanted electromedical devices, and pregnancy (87).

##### Cost

Per manufacturer, authors were not able to obtain a quote for the article.

##### Training

Current ASGE guidelines address training of capsule endoscopy (88,89), but not colon capsule training specifically. The training is incorporated into the standard 3-year fellowship, and a minimum of 20 supervised procedures are required to independently practice capsule endoscopy. For training after fellowship, a minimum 8 hour hands-on course (accredited by a national or international GI society) is required (89). Hijaz et al conducted a survey to determine the training on interpreting wireless capsule studies amongst pediatric and adult GI fellowship programs. Their findings suggest that only 4-8% of pediatrics programs have hands-on training (90). A formal curriculum is required in pediatrics capsule studies.

### Endoscopic Ultrasound

#### Description of Technology

Endoscopic ultrasound (EUS) expands endoscopic examination beyond the mucosal surface into the deeper tissues using ultrasound technology. EUS is one of the fastest growing areas within GI endoscopy. EUS was first introduced into clinical practice in 1980 (91). An early successful application was in the detection of small pancreatic neoplasms, where EUS was shown to outperform ERCP and other imaging modalities (92). Echoendoscopes can be broadly divided into two types. The radial type produces an ultrasound image, which extends outward in a plane that transects the axis of the scope. This type of device was the first to be developed and is still generally preferred for diagnostic imaging. In contrast, the linear configuration produces an ultrasound image in a plane that lies along the axis of the scope. Linear echoendoscopes are used when image-guided fine needle aspiration (FNA), core biopsy, or other intervention is required. Iterations of the basic radial and linear varieties of echoendoscope include: (1) echobronchoscopes for endobronchial ultrasound; (2) intraductal ultrasound probes for closer assessment of biliary abnormalities; (3) GI miniprobes which can be used to pass tight esophageal strictures; and (4) oblique viewing echoendoscopes designed for ERCP (93). The ESGE/ESPGHAN guidelines also recommend using a standard linear echoendoscope for children >15 kg, while endobronchial ultrasound or EUS miniprobes can be used in children <15 kg.

#### Adult and Pediatric Indications

The main diagnostic indications for EUS are well established in adults and are increasingly applied to pediatric care. For this reason, this section does not separate pediatric and adult indications but aims to highlight when pediatric literature is available. Most common indications include evaluating pancreatobiliary lesions or masses, mediastinal diseases, submucosal lesions of the gastrointestinal wall, and luminal and extraluminal malignancies. Therapeutic EUS applications are expanding. EUS-guided FNA and drainage of cystic lesions and fluid collections along the gastrointestinal tract, particularly in the pancreas, are the most common therapeutic procedures. Other innovative EUS therapeutic applications include celiac plexus neurolysis, GI bleeding, esophageal strictures, pancreatic and biliary drainage, liver biopsy, and perianal disease. The safety profile of EUS procedures has been excellent (94). Increased utilization in children will require development of child appropriate equipment, and most importantly, training opportunities for the pediatric gastroenterologists. Typical indications for EUS in pediatric patients are summarized in Table 7 (ESGE/ESPGHAN guidelines) (95).

**TABLE 7.** Typical indication for endoscopic ultrasound in pediatric patients (ESGE/ESPGHAN guidelines)

Esophagus	Stomach	Duodenum	Biliopancreatic
Congenital esophageal stenosis	Gastric duplication	Duodenal duplication	Bile duct stones
Eosinophilic esophagitis	Gastric varices		Pancreatic pseudocyst (diagnosis and treatment)
Esophageal duplications			Pancreatic disease (±FNA)

ESGE = European Society of Gastrointestinal Endoscopy; ESPGHAN = European Society for Pediatric Gastroenterology, Hepatology and Nutrition; FNA = fine needle aspiration.

##### Pancreatitis

**FIGURE 2.**
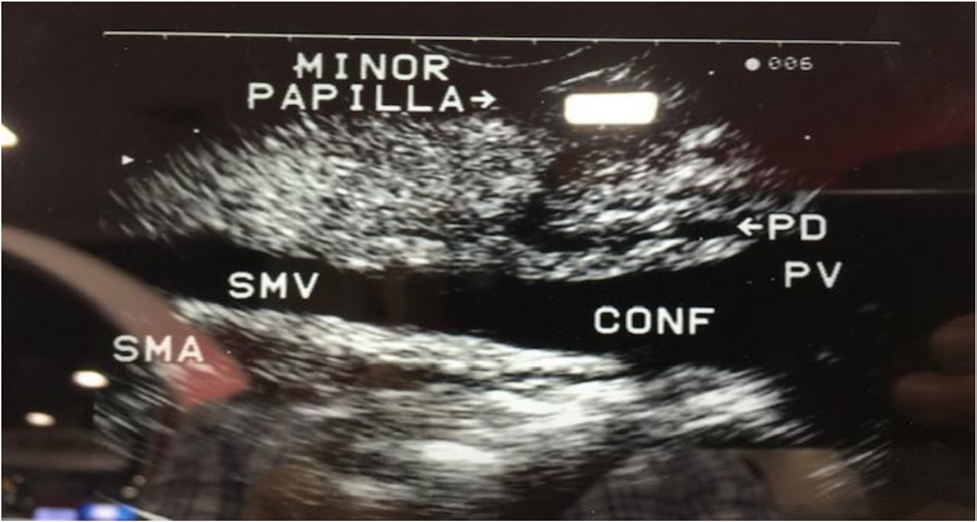
Fourteen-year-old with recurrent pancreatitis. EUS showed the PD connecting with the minor papilla diagnosis pancreas divisum. EUS = endoscopic ultrasound; PD = pancreatic duct.

**FIGURE 3.**
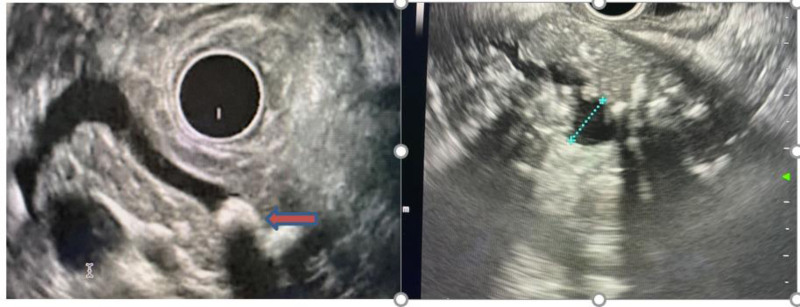
Two patients with PRSS1 mutation, show dilated pancreatic duct (blue dotted line), atrophy of parenchyma, stone (red arrow), and calcifications.

Imaging in acute pancreatitis typically involves ultrasound, magnetic resonance imaging/magnetic resonance cholangiopancreatography (MRCP), and computed tomography to identify etiology and stage disease severity. Since the EUS probe can be placed near the biliary tree and pancreas, the resulting ultrasound images can provide a more detailed examination than other modalities. EUS has been shown to identify bile duct tumors and microlithiasis (calculi <3 mm) better than ERCP or MRCP (Table 8) (96). Overall, EUS can identify the cause of “idiopathic” acute pancreatitis in two-thirds of cases (97). EUS has a sensitivity of 91% and a specificity of 86% for noncalcific pancreatitis, and EUS criteria have recently been established for the diagnosis of chronic pancreatitis and autoimmune pancreatitis (98,99). In one pediatric study of 32 children with acute recurrent pancreatitis, EUS was found to be both safe and helpful in establishing the diagnosis of chronic pancreatitis (100). Figure 2 shows an EUS diagnosis of pancreas divisum in a 14-year-old with recurrent pancreatitis. EUS can also show atrophy of the parenchyma, dilated common bile duct and calcifications commonly seen with in the setting of chronic pancreatitis (Fig. 3). US elastography may also help distinguish autoimmune pancreatitis from the circumscribed mass lesions seen in ductal adenocarcinoma (101). Although ERCP can also be utilized to investigate chronic pancreatitis, EUS offers the advantage of avoiding ERCP-associated pancreatitis, which can occur in about 10% of cases (102).

**TABLE 8.** EUS compared with ERCP and MRCP in the evaluation of acute pancreatitis

	EUS	ERCP	MRCP
Common bile duct	+++	+++	+++
Bile duct cysts	+++	+++	+++
Pancreas divisum	+++	+++	+++
Chronic pancreatitis	+++	+++	+++
Bile duct tumors	+++	+	++
Biliary microlithiasis	+++		
Sphincter of oddi dysfunction		+++	+
Risk of causing pancreatitis	No	Yes	No

ERCP = endoscopic retrograde cholangiopancreatography; EUS = endoscopic ultrasound; MRCP = magnetic resonance cholangiopancreatography.

##### Pancreatic Masses

**FIGURE 4.**
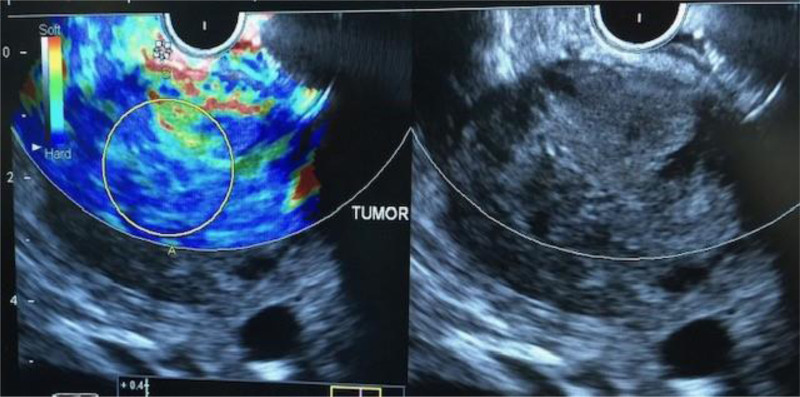
Thirteen-year-old girl with pancreatic tumor. Fine needle aspiration diagnosed a pseudopapillary tumor.

One of the most important applications for EUS is in the evaluation of patients presenting with a pancreatic mass (Fig. 4). With the advent of EUS-guided FNA, a tissue diagnosis can be made. Newer imaging techniques such as elastography and the use of contrast agents seem to improve tissue diagnosis in the setting of chronic pancreatitis, but data are limited (103–105). Contrast harmonic EUS uses intravenous microbubble contrast to visualize blood flow and better evaluate the solid components detected inside pancreatic lesions, which could be vascular cancerous lesions (106). In autoimmune pancreatitis patients, contrast harmonic EUS demonstrates a unique vascularization pattern, which can help discriminate between autoimmune pancreatitis and pancreatic cancer (107).

##### Biliary Stones

**FIGURE 5.**
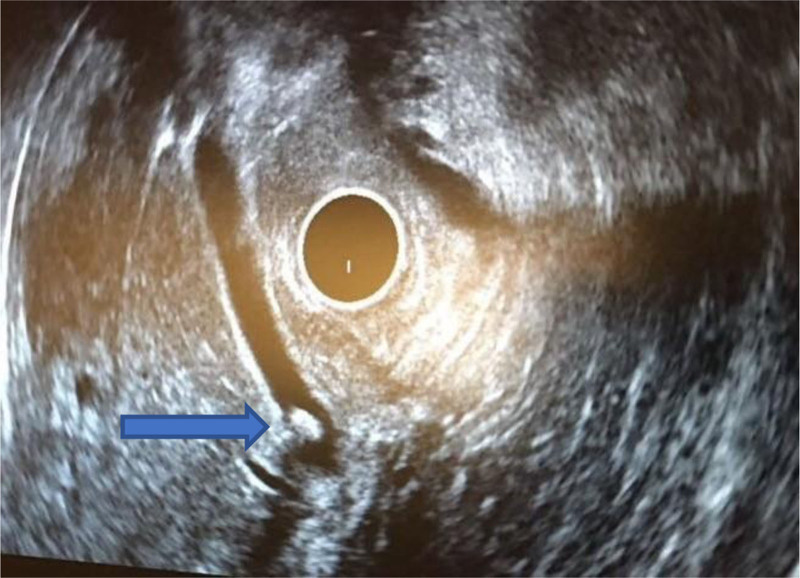
Sixteen-year-old with a stone in the common bile duct (blue arrow).

EUS has excellent overall sensitivity (94%) and specificity (95%) for the diagnosis of choledocholithiasis and improves the overall safety profile (108). For patients with intermediate probability of common bile duct stones, EUS is more sensitive than ERCP in detecting stones smaller than 4 mm (90% vs 23%). A management strategy based on EUS (with selective ERCP in patients with confirmed stones) is safer and can spare ERCP in up to 75% of patients (109–111). Sensitivity, specificity and accuracy, do not differ significantly between EUS and MRCP for the detection of choledocholithiasis (111,112). However, the sensitivity of MRCP is lower in the setting of small (<6 mm) common bile duct stones (113). Figure 5 shows a representative image of a common bile duct stone in a 16-year-old.

##### Submucosal Lesions

**FIGURE 6.**
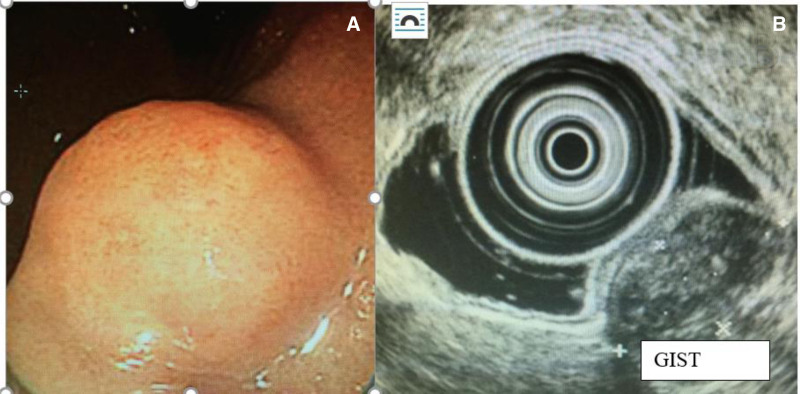
Sixteen-year-old with chronic abdominal pain. (A) Gastric submucosal lesion seen in the stomach on EGD. (B) EUS with GIST. EGD = esophagogastroduodenoscopy; EUS = endoscopic ultrasound; GIST = gastrointestinal stomal tumor.

The ability of EUS to image structures beneath the bowel mucosa makes it the ideal technique for the investigation of submucosal lesions identified during conventional endoscopy. For example, GI stromal tumors can often be identified by their origin from the muscularis propria. In the absence of diagnostic imaging findings, EUS can also be used to guide tissue sampling of suspected GI stromal tumors by FNA or core biopsy. Figure 6 shows the endoscopic findings of a submucosal lesion in a 16-year-old with chronic abdominal pain. FNA during EUS made the final diagnosis of GIST.

##### Inflammatory Bowel Disease

**FIGURE 7.**
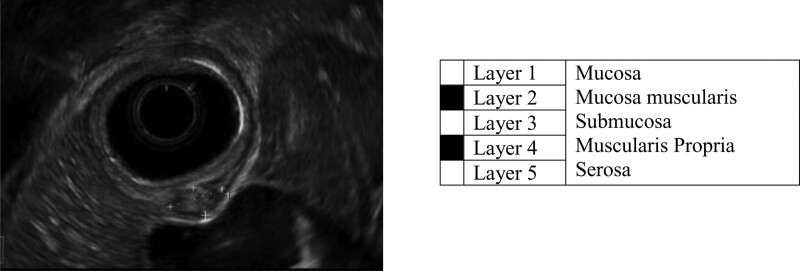
EUS showing the alternating light/dark pattern seen between the bowel wall layers (antrum). Small lesion is a submucosal mass. EUS = endoscopic ultrasound.

EUS images have a characteristic pattern alternating between light and dark (Fig. 7), which allows the endoscopist to see different layers of the bowel wall and measure bowel wall thickness. In the care of patients with IBD, this can help assess disease severity and differentiate ulcerative colitis from Crohn’s disease by distinguishing mucosal from transmural disease. EUS is not used in routine clinical practice for these purposes, but it has been shown in some studies to provide useful diagnostic information in IBD (114,115). In one adult study, IBD wall thickness was associated with active IBD, while the presence of paracolonic lymph nodes was associated with Crohn’s disease (116). EUS has also been used to help characterize small intestinal disease in conjunction with double balloon enteroscopy. In a study including 31 adult patients who underwent EUS with double balloon enteroscopy, the procedure was instrumental for therapeutic strategy in 85% of cases (117). Interestingly, the use of external ultrasound and elastography is being used increasingly in adult IBD patients while transrectal and perianal ultrasound have been shown to be helpful in the diagnosis of abscesses and fistula (118–120). The demonstrated utility of both external and transrectal ultrasound suggests that there also may be a role for EUS in children with IBD. In a pediatric study of 25 patients who underwent EUS to monitor fistula healing after seton placement, children in the EUS group were treated for a longer period due to detection of inflammatory changes (121). The use of EUS and transrectal ultrasound in the management of perianal fistulizing disease in children requires further study, but there is potential to monitor disease activity more closely with this technology.

##### Eosinophilic Esophagitis

In children with eosinophilic esophagitis (EoE), EUS has demonstrated thickened esophageal mucosa compared with children who have reflux-related disease and in control subjects, but the clinical utility of EUS in EoE management is unclear (122,123). Conventional adult and pediatric protocols for endoscopy in EoE currently do not recommend the use of EUS in EoE (124–126). However, EUS may provide useful to monitor EoE activity based on wall thickness or in the management of EoE related strictures are requires further investigation.

##### Anorectal Disease, Pyloric Stenosis, Duplications

**FIGURE 8.**
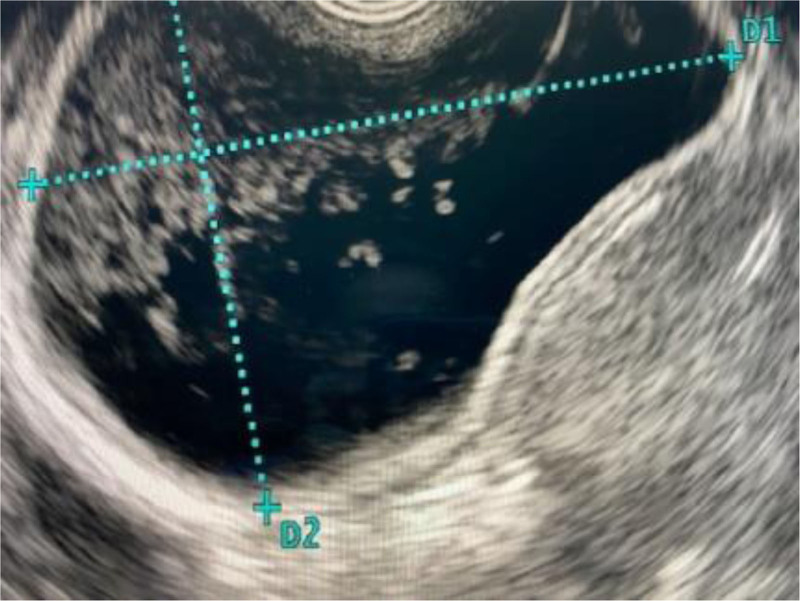
Fifteen-month-old with abdominal pain and vomiting, US and CT showed a mass in the duodenum. EGD showed compression of duodenum, and EUS showed duplication cyst with debris as shown in this image. EUS = endoscopic ultrasound.

Management of non-IBD anorectal disease, pyloric stenosis, and the diagnosis of and management of a duodenal duplication (127). EUS findings in a retrospective study of children who underwent rectal endoscopic ultrasound identified sphincter defects in two patients, including hypoplasia of the posteriolateral external sphincter and a right bisphincteric defect. The rectal EUS studies were performed without sedation in over 90% of the cases with no complications (128). In a case report in an infant with suspected pyloric stenosis, an EUS probe was guided to the pylorus using a 4.9-mm diameter endoscope and utilized to obtain high-quality imaging of the sphincter which ruled out pyloric stenosis (129). EUS has also been used to define duodenal duplications. Traditionally, intestinal duplications have been removed surgically but with EUS guidance a duplication that is remote from the biliary tree can be diagnosed and treated endoscopically by resecting the common duodenal wall (130,131). Figure 8 shows an EUS image of a 15-month-old with duodenal duplication.

##### Congenital Esophageal Stenosis

Congenital esophageal stenosis (CES) is a rare disorder, occurring in 1 per 25 000 to 50 000 live births, and can be divided into three subtypes: fibromuscular thickening (FMT), membranous web (MW), and tracheobronchial remnants (TBR). EUS with miniprobe is the only method to differentiate the subtypes (132). Typical treatment involves esophageal dilatation and/or surgical resection. The TBR subtype is associated with a lower response to dilatation as well as an increased risk of esophageal perforation during dilatation (133). Some groups, including ESGE/ESPGHAN, have therefore recommended the use of EUS to determine the CES type and reserve esophageal dilatation only for non-TBR types, while others have suggested conservative management with dilation for all types of CES as first-line management (132–135).

##### Benign Esophageal Strictures

EUS can measure the extent of esophageal stricture involvement and has been proposed as a useful adjunct to endoscopic dilatation. A prospective analysis of 27 adult and pediatric patients with esophageal strictures found that EUS could determine the depth of a stricture which in turn predicted response to dilation. Although the case number in this study was small, patients with strictures involving the submucosa (secondary to peptic injury) and muscularis (due to corrosive injury) required more dilatations than those with more superficial involvement (136). EUS has also been used to successfully create access to the distal esophagus with a needle puncture when a stricture has completely occluded the esophageal lumen (137). In situations in which it is unclear if a stricture is intrinsic or due to extrinsic compression, EUS can provide important diagnostic information with or without tissue sampling.

##### Drainage Procedures

**FIGURE 9.**
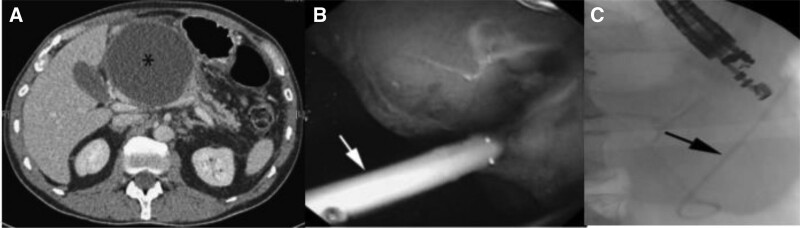
Endoscopic ultrasound in pancreatitis. (A) Computerized tomography image showing pancreatic pseudocyst (asterisk), (B) endoscopic image showing pseudocystogastrostomy plastic stent in situ (white arrow), and (C) fluoroscopic image confirming placement of pseudocytogastrotomy stent (black arrow).

Pancreatic pseudocysts are drained using a variety of techniques, including surgery, image-guided, and endoscopic-guided methods. EUS-guided drainage of pancreatic pseudocysts has become an increasingly popular technique see Figure 9, particularly in patients with portal hypertension where there may be an increased bleeding risk due to varices or liver dysfunction. Small, randomized trials comparing EUS-guided drainage with conventional endoscopic drainage found a higher success rate if EUS was utilized (138–140).

##### Liver Biopsy

EUS-guided trans gastric liver biopsy has been reported in several studies and offers several potential advantages over percutaneous biopsies, including (1) ultrasound and Doppler guidance; (2) the ability to biopsy both lobes of the liver to decrease sampling error; and (3) use of a small 19-gauge needle which could potentially minimize bleeding risk. In a prospective nonrandomized study of 110 patients who underwent the procedure, 98% obtained adequate liver tissue, and only one patient developed a subcapsular hematoma diagnosed by computed tomography scan (141). The technique may be most applicable for patients who require an upper endoscopy for another indication. EUS-guided liver biopsy has also been used successfully in pediatric patients (142).

##### Gastrointestinal Bleeding

**FIGURE 10.**
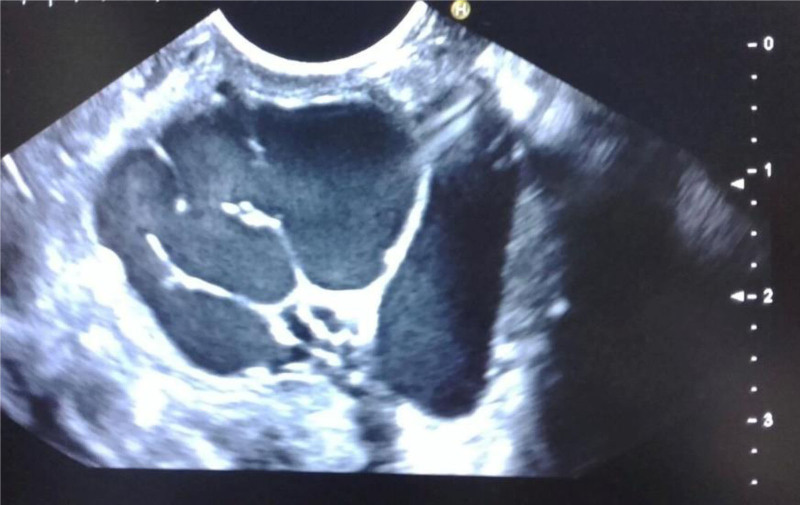
Fifteen-year-old with portal hypertension and gastric varices (with feeding vessel).

For patients with severe or obscure GI bleeding, EUS has been shown to aid in both identifying the location of the offending vessel as well as confirming that the bleeding vessel has been effectively treated. Dieulafoy lesions are arterial malformations that are extremely elusive because of their submucosal location, but EUS can identify them below the surface with ultrasound and Doppler (143). Once found, submucosal vascular malformations can be treated using conventional hemostatic methods with the aid of EUS to confirm vessel obliteration. Gauging effective treatment of GI bleeding is particularly important in life-threatening variceal bleeding. With Doppler, EUS can document the obliteration of blood flow in a bleeding vessel. Small case series have shown that the use of Doppler may prevent rebleeding and could be especially valuable for patients with recurrent or refractory bleeding (144). In a pediatric study by McKiernan and colleagues, EUS was found to be more sensitive than conventional endoscopy to detect esophageal varices. Adult studies corroborate these findings and have shown that EUS can also be used to predict variceal recurrence after therapy as well as improve the effectiveness of variceal ablation (145). Figure 10 shows gastric varices in a 15-year-old with portal hypertension.

##### Celiac Plexus Neurolysis

Celiac plexus neurolysis (CPN) has been used for the treatment of abdominal pain in pancreatic cancer and chronic pancreatitis since the 1970s (146). The technique has evolved in parallel with image guidance techniques including fluoroscopy, conventional ultrasound, computed tomography, and now EUS guidance. EUS-CPN has a low rate of complications in comparison to other image-guided CPN techniques and performs as well or better compared with computed tomography-CPN in terms of initial and medium-term reduction in analgesia requirements (147,148). EUS-CPN involves the injection of alcohol for the palliation of pancreatic carcinoma or corticosteroid for the treatment of chronic pancreatitis to the celiac plexus. Alcohol is avoided in benign disease out of concern that it may make any future surgery more difficult. A meta-analysis of EUS-CPN found a clinical success rate of over 80% in pancreatic cancer and 60% in chronic pancreatitis (149). EUS-guided endoscopic radiofrequency ablation is another technique that has been used to disrupt the celiac plexus and has been shown in one randomized study to improve pain scores compared with EUS-CPN (150).

#### Additional Information

##### Safety

Despite an increasing range of indications, complications secondary to EUS have remained low. The overall mortality rate for EUS and EUS-FNA has been reported at 0.06%, with a complication rate of under 3% (151–154). Adverse events include perforation (into the mediastinum, peritoneum, or retroperitoneum), bleeding, and bacteremia. A risk of 1%–2% of developing acute pancreatitis after EUS-FNA has been reported in multiple studies (155).

##### Cost

There are three main manufacturers of echoendoscopes, Fujifilm Endoscopy (Fujinon, Wayne, NJ), Olympus (Olympus America, Center Valley, Pa), and Pentax (Pentax of America, Montvale, NJ). The system consists of echoendoscopes generally (radial and linear) a processor, mini probes and disposables. Start-up costs are close to $400 000 and price estimates for equipment include EUS endoscopes ($44 000–113 000), probe driver ($17 290), US miniprobe (10 000), disposables ($250–$700), and EUS processors ($200 000).

##### Training

Guidelines for training have been established by several organizations for adult practice (156). A baseline competence in endoscopy is required as well as an understanding of indications, risks, and interpretation of EUS findings. To be credentialed, the ASGE recommends a minimum of 125 supervised EUS procedures for the evaluation of submucosal and mucosal abnormalities. For pancreaticobiliary evaluation, the ASGE recommends 75 supervised procedures and a minimum of 50 supervised FNA procedures to gain basic competency (157). In the United Kingdom, guidelines recommend 250 supervised procedures, including 75 to obtain competency in FNA. EUS trainers in the United Kingdom are required to undergo training of their own to insure proper instruction (158). The Forum on Canadian Endoscopic Ultrasound (FOCUS) more recently put forward a proposal for Canadian training guidelines in 2016 (159). The working group recommended a minimum of 250 supervised cases, including 50 supervised FNAs. The ESGE recommends demonstrated competence in linear ultrasound first, simulator, and live pig training for EUS-FNA when available, and a minimum of 20–30 supervised FNAs on non-pancreatic and pancreatic lesions respectively with rapid on-site cytopathological examination (160). Pediatric gastroenterologists interested in pursuing training should identify a center with experience in EUS technique and perform supervised procedures based on the above guidelines. Hands-on sessions using animal models are useful to introduce the technique and solidify skills (161). In many centers, advanced diagnostic and therapeutic procedures in pediatric patients are performed by adult gastroenterologists with advanced training. Recent ESGE/European Society for Pediatric Gastroenterology, Hepatology and Nutrition (ESPGHAN) guidelines on pediatric endoscopy makes a number of recommendations on the use of EUS in children, including that procedures be done in tertiary referral centers with experience in therapeutic endoscopy and collaboration between adult and pediatric gastroenterologists may be useful when standard echoendoscopes are utilized (95).

A summary of billing and coding for the described procedures is listed in Table 9 when the data were available.

**TABLE 9.** Reimbursement estimates when available

Name of technology	CPT codes	RVU	Nonfacility payment	Facility payment
Endoscopy with chromoendoscopy	None or as 44 799 unlisted procedure	Not established	Payer specific	Payer specific
Endoscopy with confocal laser endomicroscopy	None or as 44 799 unlisted procedure	2.29–2.96 (+0.91 report)	271	141
Optical coherence tomography	43 206, 43 252, 88 375	2.29–2.96 (+0.91 report)	271	141
Wireless motility/pH capsule	91 112	2.10	1098.71	743.49
EndoFlip	91 040	0.97	50.04	444.36
Wireless colon capsule endoscopy	91 113	Not established	Payer specific	Payer specific
Endoscopic ultrasound	45 391, 45 392, 45 341, 45 342, 43 231, 43 232, 43 237, 43 238, 43 240, 43 242, 43 253, 43 259	2.8–4.73	352.83	167.59
CPT = current procedural terminology, RVU = relative value units.				

## CONCLUSIONS

The diagnostic tools available to gastroenterologists are evolving rapidly. We summarized several technologies on the market today. Although most of these new tools are utilized for the evaluation of adult disorders, they are now evolving their roles in diagnosing pediatric diseases. Technology such as EUS is now integral to diagnosis and treatment of pediatric hepatobiliary disease and, based on consensus recommendations, chromoendoscopy should be utilized regularly to screen for metaplasia in children with long standing IBD. A pediatric gastroenterologist is uniquely trained to recognize and treat pediatric conditions and, therefore, can apply emerging technologies, often developed from an adult endoscopy perspective, to benefit the pediatric population with gastrointestinal diseases. The NASPGHAN Endoscopy Committee hopes that this document will interest the general pediatric GI community to further develop and utilize cutting edge technologies available today.

## ACKNOWLEDGMENTS

D.G.L. is the main author and editor of the article. A.M. is one of the main authors and editors of the article. I.N. wrote optical coherence tomography and significantly edited the paper for submission. C.H. wrote confocal laser endomicroscopy. K.N. wrote Endoflip section. R.A.L. wrote Endoflip section. J.K. wrote wireless motility capsule section. E.C.U., B.R.H., and R.T.P. wrote chromoendoscopy. S.M. wrote colon capsule. R.G. wrote EUS section. C.M.W. wrote confocal laser endomicroscopy and significantly edited the paper for submission. D.F. generated idea of the article and edited article.
